# Connecting DCX, COMT and FMR1 in social behavior and cognitive impairment

**DOI:** 10.1186/s12993-022-00191-7

**Published:** 2022-05-19

**Authors:** Anna Delprato, Emily Xiao, Devika Manoj

**Affiliations:** 1 Department of Research and Education, BioScience Project, Wakefield, MA 01880 USA; 2 Alexander Mackenzie High School, Richmond Hill, ON 14519 Canada; 3 Lambert High School, Suwanee, GA 30024 USA

**Keywords:** Intellectual disability, Social behavior, Neurogenesis, Hippocampus, Wnt signaling, *COMT*, *DCX*, *FMR1*

## Abstract

Genetic variants of *DCX*, *COMT* and *FMR1* have been linked to neurodevelopmental disorders related to intellectual disability and social behavior. In this systematic review we examine the roles of the *DCX*, *COMT* and *FMR1* genes in the context of hippocampal neurogenesis with respect to these disorders with the aim of identifying important hubs and signaling pathways that may bridge these conditions. Taken together our findings indicate that factors connecting *DCX*, *COMT,* and *FMR1* in intellectual disability and social behavior may converge at Wnt signaling, neuron migration, and axon and dendrite morphogenesis. Data derived from genomic research has identified a multitude of genes that are linked to brain disorders and developmental differences. Information about where and how these genes function and cooperate is lagging behind. The approach used here may help to shed light on the biological underpinnings in which key genes interface and may prove useful for the testing of specific hypotheses.

**Supplementary Information:**

The online version contains supplementary material available at 10.1186/s12993-022-00191-7.

## Introduction

The aim of this systematic review is to gain an understanding of the genetic underpinnings linking intellectual disability (ID) and social behavior in the context of three critical risk factor genes *DCX*, *COMT*, and *FMR1*. In the study by Kwan et al. [1] the authors used a method similar to ours which involved identifying signaling pathways associated with Autism Spectrum Disorder (ASD) and ID based on risk factor genes linked to these disorders that were identified in genomic studies. In this study we have started with three risk factors genes associated with a multitude of neuro-related disorders and have found through a review of the literature that they converge in Wnt signaling, neuron migration, axon, and dendrite morphogenesis. To provide further insight we use systems biology methods to investigate how these genes could interface at the molecular and cellular level. Our approach employs a literature review and an assessment of legacy RNA-Seq datasets to identify genes with correlative expression patterns to *DCX*, *COMT*, *and* *FMR1* in the developing hippocampus [2]. The gene correlates were evaluated using integrative genomics methods which include an analysis of gene set intersection [3] and functional enrichment [4]. Additional insight concerning the relationship between *DCX*, *COMT* and *FMR1* was obtained by an evaluation of protein–protein interaction (PPI) networks [5, 6].

### Hippocampal neurogenesis and Wnt signaling

In neurogenesis, neural stem cells proliferate, migrate, and differentiate into mature neurons. The production of new neurons is most active during development but continues throughout life in many species including humans [7, 8]. Hippocampal neurogenesis occurs in the subgranular zone of the dentate gyrus (DG) in a tightly regulated and sequential manner [9]. It is well established that dysregulation of hippocampal neurogenesis is linked to a variety of neurological disorders such as ASD, Fragile-X Syndrome (FXS) and ID [10–12]. This is not surprising given the role of the hippocampus in learning, long term memory, and the processing of emotional response [13].

Hippocampal neurogenesis is regulated by Wnt signaling which has been suggested as a conserved feature in both embryonic and adult neurogenesis [14–16]. The Wnt signaling pathway regulates cell fate decisions, tissue patterning, neuronal differentiation, axon outgrowth and guidance, dendrite development, synaptic function, and neuronal plasticity [17, 18].

Wnt proteins are involved in all aspects of the developing brain [19]. In neuronal development Wnt proteins bind Frizzled receptors, Tyrosine kinase receptors or the Insulin-like growth factor receptor to activate Dishevelled which results in different fates depending on the cellular context [20]. This includes gene transcription, regulation of axon and dendrite morphology and pre synaptic function via small GTPases of the Rho family, which in turn modulate neuronal polarity, dendritic spine morphology and synapses [21, 22]. The Wnt/Dishevelled axis may also proceed through a calcium signaling pathway or other pathway intermediates to modulate the guidance and branching of dendrites and axons, as well as synapse formation and remodeling [20, 23]. There is a great deal of evidence derived from genetically altered animals, cell based, and human studies supporting the role of Wnt signaling in ID and ASD [24–28]. In individuals with ASD, ID varies widely. However, in cases where [29] the two conditions coexist the *GSK3* and *CTNNB1* genes are strongly implicated [26, 30, 31]. The *CTNNB1* gene, which encodes β-catenin, is a main modulator of the canonical Wnt signaling pathway and is linked to sporadic ASD and ID [1, 29, 32]. In mice, a conditional knockout of *Ctnnb1* deleted in parvalbumin interneurons significantly impaired object recognition and social interactions and increased repetitive behaviors [24]. Moreover, data derived from large scale exome sequencing studies investigating ASD and ID have identified nonsense and missense mutations in CTNNB1 [30, 33].

### *DCX, FMR1*, and *COMT* in hippocampal neurogenesis

#### i. Role of DCX in hippocampal neurogenesis and disease

The *DCX* gene product, doublecortin, stabilizes microtubules and stimulates their polymerization to facilitate the migration of post mitotic neurons and cortical layering in the developing brain [34]. Doublecortin acts via microtubules to form a scaffold within the cell that elongates in a specific direction, altering the cytoskeleton and moving the neuron to a targeted location [35, 36]. Doublecortin is used as a neuronal differentiation and migration marker to assess the various stages of the neurogenic process in the sub granular zone (SGZ) of the hippocampus [37]. A lack of normal doublecortin affects the stability, organization and movement of microtubules which impairs their ability to move neurons [36]. Migrating neurons in the developing brain are particularly affected because they are mis-localized which disrupts connectivity resulting in neurological problems [38].

While the role of doublecortin in microtubule stabilization and neuronal migration is well established [39]. There is evidence that doublecortin is also involved in axon guidance via actin association and dendrite branching and complexity [35, 40, 41].

*Dcx* knockout mice have a simplified dendrite morphology in hippocampal pyramidal neurons [37]. Knockdown of *Dcx* in cultured rat neurons also led to a simplified dendrite morphology [15, 19]. Conversely, overexpression of doublecortin increases dendrite complexity [37]. Interestingly, daily mild stress exposure in mice altered dendrite length and complexity in doublecortin positive immature neurons of the dentate gyrus [42].

Several diseases are linked to *DCX* variants such as Isolated Lissencephaly Sequence (ILS) which is a disorder characterized by abnormal brain development that results in the brain having a smooth surface (lissencephaly) instead of normal gyri and sulci [43, 44]. This causes severe neurological issues such as ID and recurring seizures which begin in infancy. Most of the *DCX* gene mutations that cause ILS are a result of a single amino acid substitution in doublecortin producing a protein with little or no function [45].

Subcortical Band Heterotopia (SBH) is another disorder associated with mutation in the *DCX* gene [46]. This condition causes abnormal brain development that is less severe than ILS but has a similar pathology. In people with subcortical band heterotopia, some neurons that should be part of a certain region of the brain do not reach their destination [47]. Neurons stop their migration process in areas of the brain where they are not supposed to be and form band-like clusters of tissue. Male and female differences have been noted in lissencephaly and SBH related to *DCX* mutations which predominantly causes lissencephaly in hemizygous males and SBH in heterozygous females. Both males and females exhibit language impairment and epileptic seizures however cognitive ability varies between the two sexes. Males exhibit early and severe cognitive impairment whereas cognitive ability ranges from mild to severe in females [48, 49].

#### ii. Role of COMT in hippocampal neurogenesis and disease

The *COMT* gene encodes the enzyme, catechol-*O*-methyltransferase which catalyzes the transfer of a methyl group from S-adenosylmethionine to catecholamines in several neurotransmitters such as dopamine, epinephrine, and norepinephrine. This *O*-methylation results in one of the major degradative pathways of the catecholamine transmitters.

COMT has both soluble and membrane-bound isoforms and is expressed in many different tissues. The membrane bound form (MB-COMT) has a preference for brain tissue and especially the hippocampus [50]. MB-COMT is located on axons and neuron cell bodies in pre and postsynaptic structures [51]. Analyses of MB-COMT orientation with computer simulation, flow cytometry, and a cell surface enzyme assay indicates that the C-terminal catalytic domain of MB-COMT is in the extracellular space, which suggests that MB-COMT can inactivate synaptic and extrasynaptic dopamine on the surface of presynaptic and postsynaptic neurons [51].

MB-COMT is expressed by postsynaptic neurons and/or surrounding glia (Gogos et al. 1998; Schott et al. 2010; Rivett et al. 1983b; Karhunen et al. 1995a; Matsumoto et al. 2003) where it modulates synaptic dopamine levels. Dopamine levels are increased by as much as 60% in *Comt* knock-out mice [7] (e.g., Chen et al. 2004; Lebedeva et al. 2009; Grigorenko et al. 2007).

COMT localization has also been observed in dendrites [51, 52] Localization of COMT in rats using immunoelectron microscopy results in the presence of reaction product in dendritic processes and spines associated with postsynaptic membranes [50]. COMT is particularly important in the prefrontal cortex, the region of the brain associated with personality, executive function, inhibition of behaviors, abstract thinking, emotion, and working memory [53, 54]. Several studies have also demonstrated its relevance in the hippocampus [55–57] and neurogenesis [53, 58, 59]. Copy number elevation of *COMT* is associated with reduced proliferation of neural stem/progenitor cells in vitro and the migration of their progeny in the hippocampus granular layer in vivo [58] as well as hippocampal volume changes in the CA2/CA3 regions [59]. The *COMT* genotype influenced the maturation of working memory associated with problem solving and knowledge acquisition skills in both mice and humans [40, 41].

COMT and Wnt signaling are both linked to schizophrenia which has been postulated to arise from abnormal neurogenesis associated with embryonic neural stem cells [60–62]. The relationship between COMT and Wnt signaling in the context of neurogenesis may be based on dopamine regulation. The *COMT* gene has long been considered a candidate gene for schizophrenia because it degrades dopamine and individuals with schizophrenia have increased dopamine levels [63]. Wnt signaling is associated with schizophrenia, particularly via the *GSK3* gene which acts downstream of the dopamine (D2) receptor [64, 65]. Drugs that induce psychosis increase D2 receptors and drugs that are used to treat psychosis alter GSK3 signaling. GSK3 phosphorylates CTNNB1 resulting in its degradation and the down regulation of the Wnt signal [66].

*COMT* is also associated with 22q11.2 Deletion Syndrome which results from a deletion of a region of chromosome 22 that contains 30–40 genes [58]. Learning disabilities and psychiatric disturbances such as ASD, schizophrenia, and attention deficit hyperactivity disorder (ADHD) are associated with 22q11.2 Deletion Syndrome [67, 68].

Individuals with this disorder have only one copy of the *COMT* gene in each cell instead of the usual two copies making them more likely to develop neuropsychiatric disorders. *COMT* variants and dopamine levels have been linked to ASD [69]. In a study of 52 individuals diagnosed with ASD, *COMT* genotypes and dopamine levels correlated with ASD phenotype severity [69]. In another study investigating dopaminergic effects in two mouse models of ASD, differential expression of tyrosine hydroxylase (TH), the rate-limiting enzyme of catecholamine biosynthesis, was observed between the strains. There was a reduction of TH in BTBR/J mice and normal levels in *Fmr1*-KO animals. Striatal dopamine transporter expression was reduced in both strains. Interestingly, application of intranasal dopamine to *Fmr1*-KO animals alleviated their impairment of social novelty, in altered association with reduced striatal TH [70]. (https://molecularbrain.biomedcentral.com/articles/10.1186/s13041-020-00649-7).

Besides schizophrenia, ID, and ASD, COMT function in the context of dopamine regulation is also associated with addiction and depression [71–73].

#### iii. Role of *FMR1* in hippocampal neurogenesis and disease

The *FMR1* gene encodes the FMRP protein. Results from many years of research indicate that FMRP acts as a transporter carrying mRNA from the nucleus to areas of the cell where proteins are assembled [74]. Altered neurogenesis has been reported in an *Fmr1*-/-knockout mouse model. Animals displayed an increase in neuronal differentiation in the DG but no significant difference in the number of neurons added to the DG [12]. The connection between *FMR1* and Wnt signaling is supported by the finding that GSK3β, a negative regulator of Wnt signaling, is elevated in FXS animal models [65]. Correction of the increased GSK3 activity with lithium or GSK3β inhibitors in mice rescues neurobehavioral and brain morphological abnormalities [75]. Furthermore, inhibition of GSK3β is reported to improve hippocampus-dependent spatial learning tasks and restore neurogenesis in a mouse model of FXS [65].

FMRP localizes to axons and dendrites [76]. Studies involving both humans and mice support the role of FMRP expression in normal spine morphological development [77, 78]. The data obtained from human post mortem tissue derived from donors with FXS and animal models in which FMRP is underexpressed or not expressed at all indicate an increase in spine density, spine length and immature spine morphology [76, 79].

FMRP has an inhibitory effect on mRNA translation and regulates translation in pre- and post-synaptic terminals [80]. A possible explanation of the effects of FMRP in spine dynamics and morphology is by influencing local mRNA translation [81]. A trinucleotide repeat mutation in the *FMR1* gene is the underlying cause of FXS [82]. The CGG repeat disrupts gene expression and as a consequence little or no protein is produced [82]. FXS is one of the most commonly inherited forms of ID and monogenic causes of ASD [83, 84].

## Methods

### Literature review

The literature review for identifying common themes associated with *DCX*, *COMT*, and *FMR1* was performed using PubMed, Google Scholar, and the Online Mendelian Inheritance in Man database [85]. Repositories and databases were searched using keywords associations.

### Gene sets and evaluation

Microarray data were collected from the Allen Brain Database Developing Human Brain Atlas (https://human.brain-map.org/, https://human.brain-map.org/). To obtain the data, a gene search for *DCX*, *COMT*, and *FMR1* was performed. Each of these genes were used to query the atlas for correlates to the developing hippocampus.

Genes whose expression pattern correlated with DCX, COMT, and FMR1 were collected for analysis. Correlates with a range of Pearson r values from |0.7 to 1.0| were considered in the analysis (Additional file 1: Workbook S1). The rationale was to investigate genes with a similar expression pattern in order to identify correlates specific and common to DCX, COMT, and FMR1 associated with neurogenesis, Wnt signaling, ID, and social behavior.

Each gene set was evaluated using Gene Ontology (GO) enrichment via the Database for Annotation, Visualization and Integrated Discovery (DAVID, version 6.8) [86]. Gene Set overlap among the correlates for DCX, COMT, and FMR1 was assessed using Venny 2.0 [87], an online program that compares lists of items to determine the shared and unique genes.

### Network analysis

The String database (version 11.0) was used to build a protein–protein interaction network (PPI) for DCX, COMT, and FMRP [5, 88]. The network was constructed based on experimentally validated interactions using the medium confidence score of 0.4. The combined scores for the interactions are computed by combining the probabilities from the different evidence channels and corrected for the probability of randomly observing an interaction. First and 2nd shell interactions are included in the network. The network was exported from STRING and analyzed in Cytoscape (version 3.7) [6, 89]. Network clusters and enriched themes were identified with Cytoscape plugins MCODE (version 1.6.1) and ClueGo (version 2.5.7) [4, 90]. The nodes in the networks have been manually arranged for proper visibility.

## Results

To investigate how these genes may interact we performed a literature review which supported that Wnt signaling, neuron migration, and axon and dendrite morphogenesis were common factors in connecting *DCX*, *COMT,* and *FMR1* in ID and social behavior. Based on the results of the literature review, we examined RNA-Seq datasets of genes with correlating expression patterns to *DCX*, *COMT*, and *FMR1* in the developing hippocampus in order to gain further insight. GO annotation was used to identify gene correlates associated with Wnt signaling, neurogenesis, social behavior, and ID. Among the correlates, many genes are linked to Wnt signaling, neurogenesis, and ID and to a lesser extent social behavior (Tables 1, 2, 3, 4). All of the results from the GO analysis which includes biological processes, cellular localization, molecular function, as well as pathway and disease information are provided in Additional file 2: Workbook S2.

**Table 1:** Wnt pathway genes associated with DCX, COMT and FMR1 correlates in the hippocampus

DCX +	ACTB, ACTG1, ACVR1B, ARID1A, ARID1B, BCL9, CDH2, CELSR3, CSNK1E, CSNK1G1, CSNK2A1, DACT1, DCHS1, FAT4, FZD7, GNB1, GNG2, HDAC2, MYCN, PCDHB12, PCDHB14, PCDHB2, PCDHB8, PCDHB9, PPP2R5E, PYGO1, SIAH1, SMAD1, SMAD4, SMARCA4, SMARCB1, SMARCD1
DCX −	CDH19, DCHS1, GNA14, GNA15, GNG13, GNG7, KREMEN2, MYH13, MYH7B, WNT10A, WNT6, WNT9B
COMT +	BMPR1B, GNA14, ITPR3, KREMEN2, NFATC1, NFATC2 PPARD, PPP2R5A, SMARCD2, TCF7
COMT −	CELSR3, CSNK1G1, CSNK1G3, DACT1, FAT4, GSK3B, HDAC2, MAP3K7, PCDHB3, PPP2R5E, PRKCI
FMR1 +	LRP6, PPP2CA, PRKCI, SMARCA5, TBL1XR1
FMR1 −	DVL1, DVL1P1

Positive and negative associations are indicated with “+” and “−” respectively

**Table 2:** DCX, COMT and FMR1 gene correlates associated with intellectual disability

DCX +	ACTB, ACTG1, ADNP, ARID1A, ARID1B, AUTS2, BBS9, CASK, CHAMP1, CTCF, DCHS1, DYRK1A, EDC3, EFTUD2, EHMT1, EML1, EXT2, FAT4, FGD1, FOXG1, FRMD4A, FTSJ1, FXR2, GATAD2B, GNB1, IGBP1, KAT6A, KIAA2022, LMAN2L, MCPH1, NONO, OPHN1, POGZ, RBMX, RSPRY1, SETBP1, SMARCA4, SMARCB1, SMC3, SOX11, TAF2, TCF4, TTI2, TUBGCP4, ZC4H2, ZEB2, ZNF711
DCX −	BCAP31, MAP2K1
COMT +	BCAP31, CHI3L2, CLIC2, HEPACAM, PGAP3, PIGV, PPIC, SLC6A8, TECR, VWA3B
COMT −	ATP8A2, ATRX, AUTS2, BRWD3, CHAMP1, CTCF, DDX3X, EML1, FAT4, FXR2, GATAD2B, KDM6A, KIAA2022, MCPH1, MED13L, NUFIP1, PAK3, PGAP1, SETBP1, SOX5, TAF2, TCF4, TTC21B, UPF3B
FMR1 +	AMMECR1, ATRX, BRWD3, COG6, CRBN, CUL4B, FMR1, KIAA0196, KIAA1033, NIPBL, NUFIP1, NUFIP2, RAB3GAP2, RAD21, RBBP8, RPS6KA3, TBL1XR1, TDP2, TTC21B, USP9X, ZDHHC15
FMR1 −	No associated genes

Positive and negative associations are indicated with “+” and “−” respectively

**Table 3:** DCX, COMT and FMR1 gene correlates associated with social behavior

DCX +	DNAJC9, AUTS2, EIF4, EBP2
DCX −	SHANK3, ANXA7, MYH14, NPAS4
COMT +	DRD4, UCN
COMT −	KRAS, AUTS2, PCM1
FMR1 +	PCM1
FMR1 −	DVL1

Positive and negative associations are indicated with “ + ” and “-” respectively

**Table 4:** DCX, COMT and FMR1 neurogenesis related gene correlates

DCX +	AKT1, ARID1A, ARID1B, BCL11B, BZW2, CEP120, DACT1, DBN1, DCHS1, DOCK7, DPYSL2, DYNLT1, EFNB2, EPHB1, EPHB2, FAT4, FOXN4, GPSM1, IGSF9, INA, INSM1, ISLR2, KDM1A, KIAA2022, KIDINS220, NEUROD2, NGFR, OPHN1, RBM45, SEMA3A, SEMA3C, SEMA4C, SMARCA4, SMARCB1, SMARCD1, SOX11, SRGAP2, STMN1, TCF4, XRCC5
DCX −	BCL6, CHN1, CIT, GLDN, HAP1, NPAS4, NTRK1, PAX5
COMT +	BCL6, CSPG5, HAP1, METRN, MT3, NDRG2, PLXNB3, SIRT2, ZC3H12A
COMT −	ARHGEF2, BCL11B, BHLHB9, BZW2, CEP120, DACT1, EFNB2, EIF2AK4, EPHA4, EPHA7, FAT4, FBXO45, GSK3B, KIAA2022, KIDINS220, KIF2A, NEUROD6, PCM1, PRDM8, ROBO2, SEMA3A, SEMA3C, SPAST, STMN1, TCF4, XRCC5
FMR1 +	CCDC88A, EIF2AK4, HOOK3, IMPACT, PCM1, PHF10 SETX, ZEB1
FMR1 −	RFNG

Positive and negative associations are indicated with “+” and “−” respectively

The results from the analyses of gene set overlap which was performed to shed light on how these genes might interact at the molecular level, consisted of identifying common genes among the correlates for *DCX*, *COMT,* and *FMR1*. Findings indicate that there were many shared relevant genes inversely correlated with *COMT*, *DCX,* and *FMR1* expression patterns particularly in the context of ID (*CHAMP1*, *DCHS1*, *EML1*, *MCPH*, *TCF4*, *CTCF*, *FAT4*, *FXR2*, *GATAD2B*, *KIAA2022*, *SETBP1*, *TAF2*, *BCAP31*, *BRWD3*, *NUFIP1*, *ATRX*) and to a lesser extent social behavior (*AUTS2*, *PCM1*). Other shared correlating genes with relevance are linked to neurogenesis, Wnt signaling, transcription regulation, microtubule and axon related processes (Table 5 and Additional file 2: Workbook S2).

**Table 5:** Relevant shared gene correlates for DCX, COMT and FMR1

COMT +/DCX −	
BCAP31	Apoptosis, ubiquitin dependent catabolic process protein transport, X-linked mental retardation, dystonia, cerebral hypomyelination
BCL11B	Neurogenesis, axon guidance, neuron projection, transcription, splicing, methylation
GNA14	Signal transduction phospholipase C-activating dopamine receptor signaling pathway
HAP1	Synaptic transmission axonal transport, cerebellum development, cell projection organization, neurogenesis, transport along microtubules
KREMEN2	Wnt signalling
COMT −/DCX +	
AUTS2	Transcription regulation, Autism, mental retardation/ID
BZW2	Nervous system development, cell–cell adhesion, neurogenesis
CELSR3	Neuron migration, axonal fasciculation, dopaminergic serotonergic neuron axon guidance, Wnt signaling pathway
CEP120	Regulation of centrosome duplication, cerebral cortex development, neurogenesis, astral microtubule organization
CHAMP1	Protein localization to kinetochore, protein localization to microtubule, attachment of mitotic spindle microtubules to kinetochore
CSNK1G1	Endocytosis, regulation of cell shape, Wnt and Hedgehog signaling
CTCF	Transcription regulation, DNA methylation, mental retardation, Mental retardation, autosomal dominant 21
DACT1	Transcription regulation, Wnt signaling
EFNB2	Cell adhesion, axon guidance, neurogenesis
EML1	Microtubule cytoskeleton organization, epilepsy, mental retardation
FAT4	Neurogenesis, cerebral cortex development, cell adhesion, mental retardation/ID
FXR2	RNA transport, negative regulation of translation, Fragile X mental retardation
GATAD2B	Transcription, DNA methylation, mental retardation/ID
HDAC2	Transcription regulation, chromatin remodeling neuron projection and dendrite development
KIAA2022	Nervous system development, X-linked mental retardation, neurogenesis mental retardation, X-linked 98, neurite extension and migration
KIDINS220	Dendrite morphogenesis, neuron projection development, neurogenesis
MCPH1	Mitotic spindle orientation, regulation of gene expression, cerebral cortex development, mental retardation, Microcephaly 1, primary, autosomal recessive
SEMA3A	Neuron migration, axon guidance, neurogenesis
SEMA3C	Neuron migration, axon guidance, neurogenesis
SETBP1	DNA binding, Schinzel-Giedion midface retraction syndrome, mental retardation, autosomal dominant 29
STMN1	Microtubule depolymerization, mitotic spindle organization, axongenesis, neurogenesis
TAF2	Transcription regulation, mental retardation/ID Mental retardation, autosomal recessive 40
TCF4	Transcription regulation, neurogenesis, epilepsy, mental retardation, Pitt-Hopkins syndrome
XRCC5	Transcription, DNA recombination, neurogenesis
FMR1 +/COMT −	
ATRX	DNA methylation, chromatin remodeling, transcription, Mental retardation: alpha-thalassemia/mental retardation syndrome, mental retardation-hypotonic facies syndrome, X-linked 52/intellectual development disorder
BRWD3	Transcription regulation, mental retardation X-linked intellectual developmental disorder
EIF2AK4	Translation, ribosome structure and biogenesis, learning and long-term memory
NUFIP1	RNA processing, transcription fragile X mental retardation-interacting protein 1
PCM1	Neuron migration, microtubule organization and anchoring, social behavior, negative regulation of neurogenesis
TTC21B	Transcription regulator, smoothened signaling pathway regulation
FMR1 −/COMT +	
GAS6	Dendritic cell differentiation, apoptosis
KIF19	Axon, microtubule depolymeriation
NDUFS7	Synapse, neuron projection

Positive and negative correlative gene expression patterns are indicated with “+” and “−” respectively

The majority of ID related genes are shared between *DCX* positive and *COMT* negative correlates. A possible explanation could be related to the roles of *COMT* and *DCX* and their effects on brain structure and neurogenesis. In patients with schizophrenia the *COMT* *Val* allele is associated with smaller temporal and frontal brain areas [91] and as described in the Introduction, *DCX* variants cause severe lamination defects in the cortical and hippocampus regions [92]. In addition, there is supporting evidence that increased dopamine neurotransmission stimulates neurogenesis [93, 94].

The shared genes between *COMT* and *FMR1* are also inversely correlated and are associated with similar relevant themes (Table 5). There were no relevant genes common between *FMR1* and *DCX*.

**Fig. 1:**
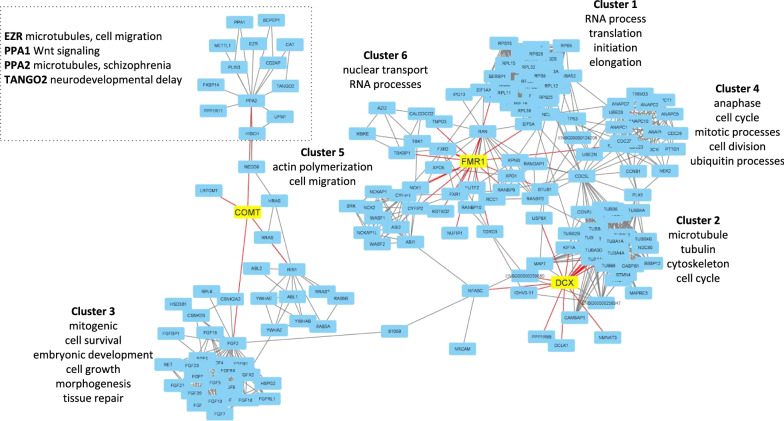
DCX, COMT, and FMRP PPI network. A topological evaluation of PPI networks of DCX, COMT, and FMRP indicates that DCX and FMRP networks are highly interconnected, whereas the COMT network is peripherally associated with the DCX network through second shell interactions. Red lines represent first shell interactions which are proteins directly associated with DCX, COMT, and FMRP. Gray lines indicate second shell interactions which are proteins linked with the other proteins in the first shell i.e., not DCX, COMT, or FMR1P

To gain further insight, an analysis of protein–protein interaction networks of experimentally validated interactions for *DCX*, *COMT*, and *FMRP* was performed. An assessment of network topology and connectivity indicates that the individual PPI networks for these genes connect (Fig. 1, Additional file 3: Workbook S3).

The DCX and FMRP networks are more highly interconnected via proteins associated with RNA binding and cell cycle such as FXR1/2 and CYFIP2, whereas the COMT network is linked to the DCX and FMRP networks via the neurotrophic factor S100B which enhances hippocampal neurogenesis in rodent models, as well as the microtubule associated proteins MAPT, which promotes microtubule assembly and stability and TUBA1A which is a fundamental component of microtubules [95–98].

Assignment of over-represented themes based on GO and pathway analysis of the PPI network modules are: Module 1. RNA process translation initiation elongation, Module 2. Microtubule tubulin cytoskeleton cell cycle, Module 3. Mitogenic cell survival embryonic development cell growth morphogenesis tissue repair, Module 4. Anaphase cell cycle mitotic, cell division and ubiquitin processes, Module 5. Actin polymerization cell migration, Module 6. Nuclear transport, RNA processes (Fig. 1).

**Fig. 2:**
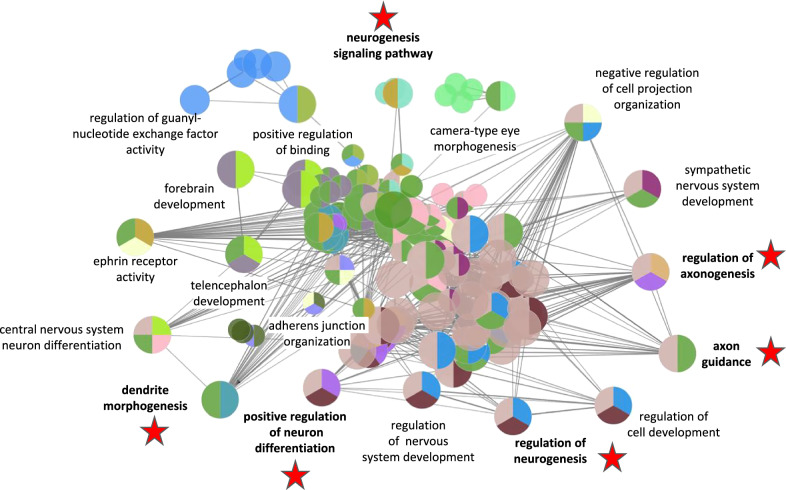
Functional enrichment of DCX, COMT, and FMRP correlates linked to neurogenesis Neurogenesis related gene correlates were further evaluated for functional associations in the context of biological process and pathway integration. Enriched themes include axon guidance, axiogenesis, cell projection and dendritic processes. Red stars indicate functional enrichment themes with particular relevance to the study. Colors are chosen arbitrarily for distinction and to depict overlap.

An analysis of neurogenesis related genes from the *DCX*, *COMT*, and *FMR1* correlates in the context of enriched functional categories results in thirty-two groups and within those groups one hundred and ninety-one GO annotations. Among the categories there are many related to axon, dendrite, and neuron processes as well as several other relevant classifications (Fig. 2 and Additional file 4: Workbook S4).

## Conclusions

Polymorphisms in the *DCX*, *COMT*, and *FMR1* genes are associated with severe and diverse brain development and neuropsychiatric disorders. Each of these genes has been linked to ID and social behavior. To investigate how these genes may interact we performed a literature review which pointed to Wnt signaling, neuron migration, and axon and dendrite morphogenesis as common factors.

Based on the results from the literature review, we analyzed gene expression patterns in the developing hippocampus to gain additional support and insight into the relationship between these genes in the context of identifying molecular interactions and signaling pathways that may connect them. The findings from these analyses support the results obtained from the literature review and provide useful information for follow up studies.

## Supplementary Information

**Additional file 1: Workbook S1.** *DCX*, *COMT*, and *FMR1* hippocampal gene correlates.

**Additional file 2: Workbook S2.** Gene Ontology analysis of *DCX*, *COMT*, and *FMR1* hippocampal gene correlates.

**Additional file 3: Workbook S3.** DCX, COMT, and FMRP protein–protein interaction network.

**Additional file 4: Workbook S4.** *DCX*, *COMT*, and *FMR1* functional enrichment categories for neurogenesis genes.

## Data Availability

All data are provided with the manuscript.

## References

[1] Kwan V, Unda BK, Singh KK (2016). Wnt signaling networks in autism spectrum disorder and intellectual disability. J Neurodev Disord.

[2] Jones AR, Overly CC, Sunkin SM (2009). The Allen Brain Atlas: 5 years and beyond. Nat Rev Neurosci.

[3] Conway JR, Lex A, Gehlenborg N (2017). UpSetR: an R package for the visualization of intersecting sets and their properties. Bioinformatics.

[4] Bindea G, Mlecnik B, Hackl H, Charoentong P, Tosolini M, Kirilovsky A (2009). ClueGO: a Cytoscape plug-in to decipher functionally grouped gene ontology and pathway annotation networks. Bioinformatics.

[5] Szklarczyk D, Gable AL, Lyon D, Junge A, Wyder S, Huerta-Cepas J (2019). STRING v11: protein-protein association networks with increased coverage, supporting functional discovery in genome-wide experimental datasets. Nucleic Acids Res.

[6] Shannon P, Markiel A, Ozier O, Baliga NS, Wang JT, Ramage D (2003). Cytoscape: a software environment for integrated models of biomolecular interaction networks. Genome Res.

[7] Lepousez G, Nissant A, Lledo P-M (2015). Adult neurogenesis and the future of the rejuvenating brain circuits. Neuron.

[8] Kumar A, Pareek V, Faiq MA, Ghosh SK, Kumari C (2019). Adult neurogenesis in humans: a review of basic concepts, history, current research, and clinical implications. Innov Clin Neurosci.

[9] Zhong S, Ding W, Sun L, Lu Y, Dong H, Fan X (2020). Decoding the development of the human hippocampus. Nature.

[10] Packer A (2016). Neocortical neurogenesis and the etiology of autism spectrum disorder. Neurosci Biobehav Rev.

[11] Pons-Espinal M, de Lagran MM, Dierssen M (2013). Functional implications of hippocampal adult neurogenesis in intellectual disabilities. Amino Acids.

[12] Eadie BD, Zhang WN, Boehme F, Gil-Mohapel J, Kainer L, Simpson JM (2009). Fmr1 knockout mice show reduced anxiety and alterations in neurogenesis that are specific to the ventral dentate gyrus. Neurobiol Dis.

[13] Andersen P, Morris R, Amaral D, Bliss T, O’Keefe J (2006). The hippocampus book.

[14] Arredondo SB, Valenzuela-Bezanilla D, Mardones MD, Varela-Nallar L (2020). Role of wnt signaling in adult hippocampal neurogenesis in health and disease. Front Cell Dev Biol.

[15] Lie D-C, Colamarino SA, Song H-J, Désiré L, Mira H, Consiglio A (2005). Wnt signalling regulates adult hippocampal neurogenesis. Nature.

[16] Lee SM, Tole S, Grove E, McMahon AP (2000). A local Wnt-3a signal is required for development of the mammalian hippocampus. Development.

[17] Rosso SB, Inestrosa NC (2013). WNT signaling in neuronal maturation and synaptogenesis. Front Cell Neurosci.

[18] He C-W, Liao C-P, Pan C-L (2018). Wnt signalling in the development of axon, dendrites and synapses. Open Biol.

[19] Mulligan KA, Cheyette BNR (2012). Wnt signaling in vertebrate neural development and function. J Neuroimmune Pharmacol.

[20] Rao TP, Kühl M (2010). An updated overview on Wnt signaling pathways: a prelude for more. Circ Res.

[21] Esufali S, Bapat B (2004). Cross-talk between Rac1 GTPase and dysregulated Wnt signaling pathway leads to cellular redistribution of beta-catenin and TCF/LEF-mediated transcriptional activation. Oncogene.

[22] Schlessinger K, Hall A, Tolwinski N (2009). Wnt signaling pathways meet Rho GTPases. Genes Dev.

[23] Komiya Y, Habas R (2008). Wnt signal transduction pathways. Organogenesis.

[24] Dong F, Jiang J, McSweeney C, Zou D, Liu L, Mao Y (2016). Deletion of CTNNB1 in inhibitory circuitry contributes to autism-associated behavioral defects. Hum Mol Genet.

[25] Takata A, Ionita-Laza I, Gogos JA, Xu B, Karayiorgou M (2016). De novo synonymous mutations in regulatory elements contribute to the genetic etiology of autism and schizophrenia. Neuron.

[26] Kuechler A, Willemsen MH, Albrecht B, Bacino CA, Bartholomew DW, van Bokhoven H (2015). De novo mutations in beta-catenin (CTNNB1) appear to be a frequent cause of intellectual disability: expanding the mutational and clinical spectrum. Hum Genet.

[27] Snijders Blok L, Madsen E, Juusola J, Gilissen C, Baralle D, Reijnders MRF (2015). Mutations in DDX3X are a common cause of unexplained intellectual disability with gender-specific effects on Wnt signaling. Am J Hum Genet.

[28] Kumar S, Reynolds K, Ji Y, Gu R, Rai S, Zhou CJ (2019). Impaired neurodevelopmental pathways in autism spectrum disorder: a review of signaling mechanisms and crosstalk. J Neurodev Disord.

[29] Bae SM, Hong JY (2018). The Wnt signaling pathway and related therapeutic drugs in autism spectrum disorder. Clin Psychopharmacol Neurosci.

[30] de Ligt J, Willemsen MH, van Bon BWM, Kleefstra T, Yntema HG, Kroes T (2012). Diagnostic exome sequencing in persons with severe intellectual disability. N Engl J Med.

[31] Caracci MO, Ávila ME, De Ferrari GV (2016). Synaptic Wnt/GSK3β signaling hub in autism. Neural Plast.

[32] Krumm N, O’Roak BJ, Shendure J, Eichler EE (2014). A de novo convergence of autism genetics and molecular neuroscience. Trends Neurosci.

[33] O’Roak BJ, Vives L, Girirajan S, Karakoc E, Krumm N, Coe BP (2012). Sporadic autism exomes reveal a highly interconnected protein network of de novo mutations. Nature.

[34] Tint I, Jean D, Baas PW, Black MM (2009). Doublecortin associates with microtubules preferentially in regions of the axon displaying actin-rich protrusive structures. J Neurosci.

[35] Fu X, Brown KJ, Yap CC, Winckler B, Jaiswal JK, Liu JS (2013). Doublecortin (Dcx) family proteins regulate filamentous actin structure in developing neurons. J Neurosci.

[36] Ayanlaja AA, Xiong Y, Gao Y, Ji G, Tang C, Abdikani Abdullah Z (2017). Distinct features of doublecortin as a marker of neuronal migration and its implications in cancer cell mobility. Front Mol Neurosci.

[37] Cohen D, Segal M, Reiner O (2008). Doublecortin supports the development of dendritic arbors in primary hippocampal neurons. Dev Neurosci.

[38] Guarnieri FC, de Chevigny A, Falace A, Cardoso C (2018). Disorders of neurogenesis and cortical development. Dialogues Clin Neurosci.

[39] Gleeson JG, Lin PT, Flanagan LA, Walsh CA (1999). Doublecortin is a microtubule-associated protein and is expressed widely by migrating neurons. Neuron.

[40] Nosten-Bertrand M, Kappeler C, Dinocourt C, Denis C, Germain J, Phan Dinh Tuy F (2008). Epilepsy in Dcx knockout mice associated with discrete lamination defects and enhanced excitability in the hippocampus. PLoS ONE.

[41] Sánchez-Huertas C, Herrera E (2021). With the permission of microtubules: an updated overview on microtubule function during axon pathfinding. Front Mol Neurosci.

[42] Dioli C, Patrício P, Sousa N, Kokras N, Dalla C, Guerreiro S (2019). Chronic stress triggers divergent dendritic alterations in immature neurons of the adult hippocampus, depending on their ultimate terminal fields. Transl Psychiatry.

[43] Dobyns WB (2010). The clinical patterns and molecular genetics of lissencephaly and subcortical band heterotopia. Epilepsia.

[44] Bahi-Buisson N, Souville I, Fourniol FJ, Toussaint A, Moores CA, Houdusse A (2013). New insights into genotype-phenotype correlations for the doublecortin-related lissencephaly spectrum. Brain.

[45] Sapir T, Horesh D, Caspi M, Atlas R, Burgess HA, Wolf SG (2000). Doublecortin mutations cluster in evolutionarily conserved functional domains. Hum Mol Genet.

[46] Matsumoto N, Leventer RJ, Kuc JA, Mewborn SK, Dudlicek LL, Ramocki MB (2001). Mutation analysis of the DCX gene and genotype/phenotype correlation in subcortical band heterotopia. Eur J Hum Genet.

[47] Haverfield EV, Whited AJ, Petras KS, Dobyns WB, Das S (2009). Intragenic deletions and duplications of the LIS1 and DCX genes: a major disease-causing mechanism in lissencephaly and subcortical band heterotopia. Eur J Hum Genet.

[48] Hehr U, Uyanik G, Aigner L, Couillard-Despres S, Winkler J, Adam MP, Ardinger HH, Pagon RA, Wallace SE, Bean LJ, Mefford HC (1993). DCX-related disorders. GeneReviews^®^.

[49] Shmueli O, Gdalyahu A, Sorokina K, Nevo E, Avivi A, Reiner O (2001). DCX in PC12 cells: CREB-mediated transcription and neurite outgrowth. Hum Mol Genet.

[50] Karhunen T, Tilgmann C, Ulmanen I, Panula P (1995). Catechol-O-methyltransferase (COMT) in rat brain: immunoelectron microscopic study with an antiserum against rat recombinant COMT protein. Neurosci Lett.

[51] Chen J, Song J, Yuan P, Tian Q, Ji Y, Ren-Patterson R (2011). Orientation and cellular distribution of membrane-bound catechol-O-methyltransferase in cortical neurons: implications for drug development. J Biol Chem.

[52] Matsumoto M, Weickert CS, Akil M, Lipska BK, Hyde TM, Herman MM (2003). Catechol O-methyltransferase mRNA expression in human and rat brain: evidence for a role in cortical neuronal function. Neuroscience.

[53] Dumontheil I, Roggeman C, Ziermans T, Peyrard-Janvid M, Matsson H, Kere J (2011). Influence of the COMT genotype on working memory and brain activity changes during development. Biol Psychiatry.

[54] Bishop SJ, Cohen JD, Fossella J, Casey BJ, Farah MJ (2006). COMT genotype influences prefrontal response to emotional distraction. Cogn Affect Behav Neurosci.

[55] Laatikainen LM, Sharp T, Bannerman DM, Harrison PJ, Tunbridge EM (2012). Modulation of hippocampal dopamine metabolism and hippocampal-dependent cognitive function by catechol-O-methyltransferase inhibition. J Psychopharmacol.

[56] van Rooij SJH, Stevens JS, Ely TD, Fani N, Smith AK, Kerley KA (2016). Childhood trauma and COMT genotype interact to increase hippocampal activation in resilient individuals. Front Psychiatry.

[57] Taylor WD, Züchner S, Payne ME, Messer DF, Doty TJ, MacFall JR (2007). The COMT Val158Met polymorphism and temporal lobe morphometry in healthy adults. Psychiatry Res.

[58] Boku S, Izumi T, Abe S, Takahashi T, Nishi A, Nomaru H (2018). Copy number elevation of 22q11.2 genes arrests the developmental maturation of working memory capacity and adult hippocampal neurogenesis. Mol Psychiatry.

[59] Rabl U, Meyer BM, Diers K, Bartova L, Berger A, Mandorfer D (2014). Additive gene-environment effects on hippocampal structure in healthy humans. J Neurosci.

[60] Miyaoka T, Seno H, Ishino H (1999). Increased expression of Wnt-1 in schizophrenic brains. Schizophr Res.

[61] Freyberg Z, Ferrando SJ, Javitch JA (2010). Roles of the Akt/GSK-3 and Wnt signaling pathways in schizophrenia and antipsychotic drug action. Am J Psychiatry.

[62] Iannitelli A, Quartini A, Tirassa P, Bersani G (2017). Schizophrenia and neurogenesis: a stem cell approach. Neurosci Biobehav Rev.

[63] Ross CA, Margolis RL, Reading SAJ, Pletnikov M, Coyle JT (2006). Neurobiology of schizophrenia. Neuron.

[64] Jope RS, Roh M-S (2006). Glycogen synthase kinase-3 (GSK3) in psychiatric diseases and therapeutic interventions. Curr Drug Targets.

[65] Guo W, Murthy AC, Zhang L, Johnson EB, Schaller EG, Allan AM (2012). Inhibition of GSK3β improves hippocampus-dependent learning and rescues neurogenesis in a mouse model of fragile X syndrome. Hum Mol Genet.

[66] Li X, Jope RS (2010). Is glycogen synthase kinase-3 a central modulator in mood regulation?. Neuropsychopharmacology.

[67] DiGeorge syndrome (22q11 deletion)—NHS—NHS. https://www.nhs.uk/conditions/digeorge-syndrome/. Accessed 19 Dec 2021.

[68] OMIM Entry 88400—Digeorge syndrome; DGS. https://www.omim.org/entry/188400?search=digeorge%20syndrome&highlight=%28syndrome%7Csyndromic%29%20digeorge. Accessed 19 Dec 2021.

[69] Esmaiel NN, Ashaat EA, Mosaad R, Fayez A, Ibrahim M, Abdallah ZY (2020). The potential impact of COMT gene variants on dopamine regulation and phenotypic traits of ASD patients. Behav Brain Res.

[70] Chao OY, Pathak SS, Zhang H, Dunaway N, Li J-S, Mattern C (2020). Altered dopaminergic pathways and therapeutic effects of intranasal dopamine in two distinct mouse models of autism. Mol Brain.

[71] Lohoff FW, Weller AE, Bloch PJ, Nall AH, Ferraro TN, Kampman KM (2008). Association between the catechol-O-methyltransferase Val158Met polymorphism and cocaine dependence. Neuropsychopharmacology.

[72] Guillot CR, Fanning JR, Liang T, Berman ME (2015). COMT associations with disordered gambling and drinking measures. J Gambl Stud.

[73] Funke B, Malhotra AK, Finn CT, Plocik AM, Lake SL, Lencz T (2005). COMT genetic variation confers risk for psychotic and affective disorders: a case control study. Behav Brain Funct.

[74] Bardoni B, Capovilla M, Lalli E (2017). Modeling Fragile X syndrome in neurogenesis: an unexpected phenotype and a novel tool for future therapies. Neurogenesis.

[75] Min WW, Yuskaitis CJ, Yan Q, Sikorski C, Chen S, Jope RS (2009). Elevated glycogen synthase kinase-3 activity in Fragile X mice: key metabolic regulator with evidence for treatment potential. Neuropharmacology.

[76] Antar LN, Li C, Zhang H, Carroll RC, Bassell GJ (2006). Local functions for FMRP in axon growth cone motility and activity-dependent regulation of filopodia and spine synapses. Mol Cell Neurosci.

[77] Nimchinsky EA, Oberlander AM, Svoboda K (2001). Abnormal development of dendritic spines in FMR1 knock-out mice. J Neurosci.

[78] Comery TA, Harris JB, Willems PJ, Oostra BA, Irwin SA, Weiler IJ (1997). Abnormal dendritic spines in fragile X knockout mice: maturation and pruning deficits. Proc Natl Acad Sci USA.

[79] He CX, Portera-Cailliau C (2013). The trouble with spines in fragile X syndrome: density, maturity and plasticity. Neuroscience.

[80] Li Z, Zhang Y, Ku L, Wilkinson KD, Warren ST, Feng Y (2001). The fragile X mental retardation protein inhibits translation via interacting with mRNA. Nucleic Acids Res.

[81] Banerjee A, Ifrim MF, Valdez AN, Raj N, Bassell GJ (2018). Aberrant RNA translation in fragile X syndrome: From FMRP mechanisms to emerging therapeutic strategies. Brain Res.

[82] Jin P, Warren ST (2000). Understanding the molecular basis of fragile X syndrome. Hum Mol Genet.

[83] Kazdoba TM, Leach PT, Silverman JL, Crawley JN (2014). Modeling fragile X syndrome in the Fmr1 knockout mouse. Intractable Rare Dis Res.

[84] Dahlhaus R (2018). Of men and mice: modeling the fragile X syndrome. Front Mol Neurosci.

[85] Hamosh A, Scott AF, Amberger JS, Bocchini CA, McKusick VA (2005). Online mendelian inheritance in man (OMIM), a knowledgebase of human genes and genetic disorders. Nucleic Acids Res.

[86] Huang DW, Sherman BT, Zheng X, Yang J, Imamichi T, Stephens R (2009). Extracting biological meaning from large gene lists with DAVID. Curr Protoc Bioinform.

[87] Venny 2.1.0. https://bioinfogp.cnb.csic.es/tools/venny/. Accessed 7 Sep 2020.

[88] STRING: functional protein association networks. https://string-db.org/. Accessed 7 Sep 2020.

[89] Cytoscape: an open source platform for complex network analysis and visualization. https://cytoscape.org/. Accessed 7 Sep 2020.

[90] Bader GD, Hogue CWV (2003). An automated method for finding molecular complexes in large protein interaction networks. BMC Bioinform.

[91] McIntosh AM, Baig BJ, Hall J, Job D, Whalley HC, Lymer GKS (2007). Relationship of catechol-O-methyltransferase variants to brain structure and function in a population at high risk of psychosis. Biol Psychiatry.

[92] Corbo JC, Deuel TA, Long JM, LaPorte P, Tsai E, Wynshaw-Boris A (2002). Doublecortin is required in mice for lamination of the hippocampus but not the neocortex. J Neurosci.

[93] Salvi R, Steigleder T, Schlachetzki JCM, Waldmann E, Schwab S, Winner B (2016). Distinct effects of chronic dopaminergic stimulation on hippocampal neurogenesis and striatal doublecortin expression in adult mice. Front Neurosci.

[94] Borta A, Höglinger GU (2007). Dopamine and adult neurogenesis. J Neurochem.

[95] Kleindienst A, Grünbeck F, Buslei R, Emtmann I, Buchfelder M (2013). Intraperitoneal treatment with S100B enhances hippocampal neurogenesis in juvenile mice and after experimental brain injury. Acta Neurochir.

[96] Komuro Y, Xu G, Bhaskar K, Lamb BT (2015). Human tau expression reduces adult neurogenesis in a mouse model of tauopathy. Neurobiol Aging.

[97] Hebebrand M, Hüffmeier U, Trollmann R, Hehr U, Uebe S, Ekici AB (2019). The mutational and phenotypic spectrum of TUBA1A-associated tubulinopathy. Orphanet J Rare Dis.

[98] Wang J-Z, Liu F (2008). Microtubule-associated protein tau in development, degeneration and protection of neurons. Prog Neurobiol.

